# Investigation of choroidal structure changes after intravitreal anti-VEGF therapy for retinal vein occlusion

**DOI:** 10.1007/s00417-024-06562-2

**Published:** 2024-07-22

**Authors:** Erdem Dursun, Baki Derhem, Seval Çobanoğlu, Tevfik Oğurel

**Affiliations:** 1 Faculty of Medicine, Ophthalmology Department, Kırıkkale University, Seyrantepe Mh. Asude Kent Sitesi C Blok no:22 Yahsihan, Kırıkkale, Turkey; 2 Faculty of Medicine, Family Medicine Department, Kırıkkale University, Kırıkkale, Turkey

**Keywords:** Retinal vein occlusion, Choroid thickness, Central macular thickness, Vascular endothelial growth factor, Choroid vascularity index

## Abstract

**Background:**

We aimed to investigate the effect of retinal vein occlusion (RVO) on the posterior segment structures of the eye and its changes with intravitreal anti-Vascular Endothelial Growth Factor (VEGF) treatment.

**Methods:**

This prospective longitudinal study included 29 eyes of 29 patients with RVO (17 males and 12 females) followed for 6 months. The best corrected visual acuity (BCVA), macula, choroid ticknesses and choroidal vascularity index (CVI) obtained by spectral-domain optical coherence tomography were recorded at baseline and the first, third, and sixth months after the first injection. Results were compared with fellow eyes (non-affected eyes) and age- and sex-matched controls.

**Results:**

BCVA increased significantly in the 6th month, more in the first month of injection (*p* < 0.05 for each). Central macular tickness, subfoveal choroid tickness, stromal and total area of choroid decreased significantly after injection (*p* < 0.05 for each). CVI values increased significantly, especially in the 1st month after injection (*p* < 0.05 for each). In eyes with Branch RVO, there was a significant decrease in the macular thickness of the occlusive areas with treatment, while there was no statistically significant change in the non-occlusive macular thickness.

**Conclusion:**

Observation of changes in choroidal structure may be useful to assess the activity of RVO and predict the efficacy of anti-VEGF therapy.

## Key messages

***What is known***

• Retinal vein occlusion is one of the most common retinal vascular diseases after diabetic retinopathy.

• The main role of choroid is to supply nutrients to the outer retina, but the hemodynamics of the cho­roidal circulation is not yet fully understood.

***What is new***

• Choroidal vascularity index was lower in retinal vein occlusion compared to controls, but increased signifi­cantly with anti-vascular endothelial growth factor treatment, especially at 1 month after injection.

• The choroidal thickening observed in retinal vein occlusion appears to result from stromal edema, possibly triggered by elevated intraocular vascular endothelial growth factor levels, rather than direct vascular dilatation.

## Introduction

Retinal vein occlusion (RVO) is a prevalent retinal vascular disease that occurs after diabetic retinopathy [1]. Branch retinal vein occlusion (BRVO) occurs significantly more often than central retinal vein occlusion (CRVO), with a prevalence four to six times higher. RVO can also be ischemic or non-ischemic [2]. The prevalence in RVO has been reported to be 0,5% over 40 years of age [3, 4]. Systemic conditions such as hypertension (HT), diabetes mellitus (DM), hyperlipidemia (HL), atherosclerosis and hyperviscosity syndrome, and ocular diseases such as glaucoma are considered in the etiology [5, 6].

In patients with RVO, Vascular Endothelial Growth Factor (VEGF) increases vascular permeability by increasing phosphorylation of tight junction proteins and is therefore an important mediator of blood-retinal barrier degradation that leads to vascular leakage and macular edema. Therapy that inhibits VEGF is an effective therapeutic modality targeting the underlying pathogenesis of macular edema in RVO and anti-VEGF intravitreal therapy has become the standard of care for treating this disease [7].

The main role of the choroid is to provide nutrients to the outer retina. The role of the choroid in the pathogenesis of ocular diseases is well established, however the hemodynamics of the choroidal circulation has not been fully understood yet. Studies on the choroid in RVO are limited by contradictory result in changes of choroidal thicknesses and structure of choriocapillaris [8–10].

In this study, we aimed to examine the structural changes in the choroid in RVO patients administered intravitreal anti-VEGF and to evaluate whether these changes would be useful in the follow-up of the patients.

## Subjects and methods

**Fig. 1:**
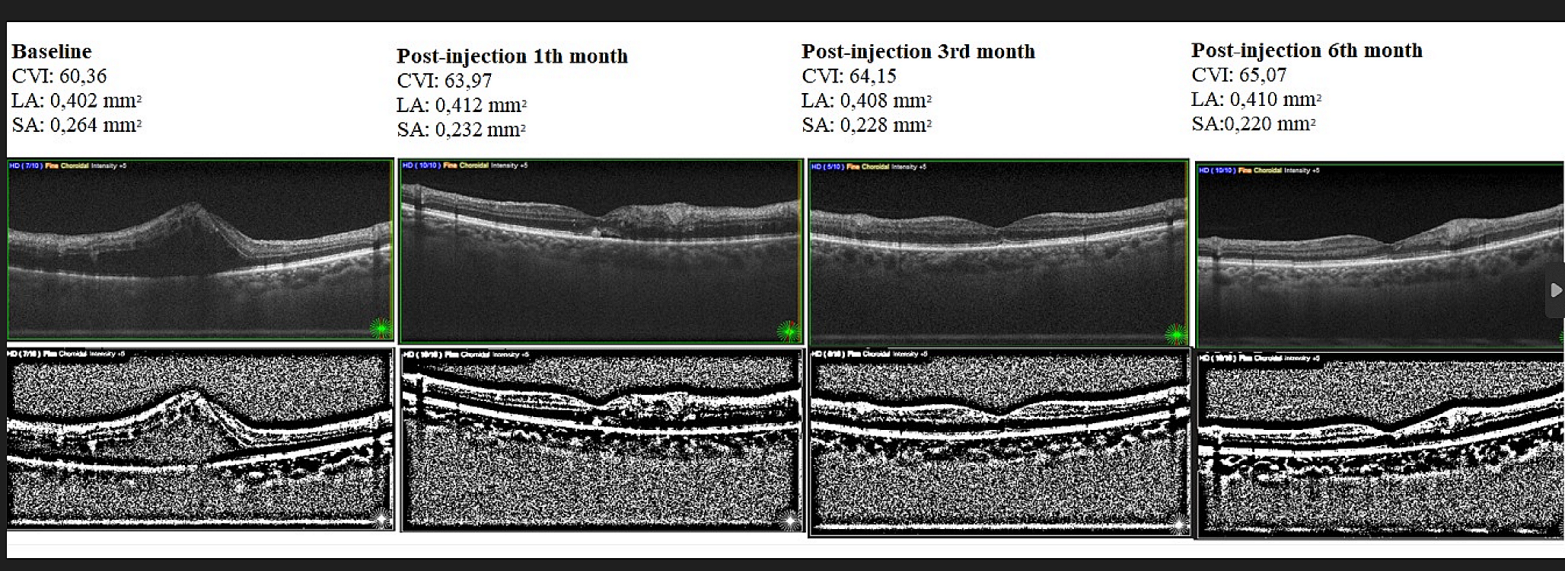
The OCT B-Scans in the image show the changes in the stromal and luminal area of the choroid with treatment

Ethical approval for the study was received from the Kırıkkale University Clinical Research Ethics Committee. RVO patients who applied to the Kırıkkale University Faculty of Medicine Department of Ophthalmology between January 2022 and December 2022 were followed prospectively. The diagnosis of RVO was made after detailed history and fundus examination, supported by Optical Coherence Tomography (OCT) and Fundus Fluorescein Angiography (FFA). CRVO was defined as retinal hemorrhage, telangiectatic capillary bed and a dilated venous system in all 4 quadrants. BRVO was defined as retinal hemorrhage or other biomicroscopic evidence of RVO in less than all 4 quadrants. In patients diagnosed with BRVO, occluded and non-occluded areas were evaluated separately. Ischemic RVO is defined as the presence of more than 10 disc areas of retinal non-perfused area on FFA. Anti-VEGF therapy was administered to all patients with macular oedema in accordance with The Royal College of Ophthalmologists Guidelines on retinal vein occlusions version 2022. All participants received 3 consecutive monthly bevacizumab injections, and followed by a pro re nata regimen (as needed after 3 injection) (see Fig. 1).

Gender, age, systemic diseases, type of RVO, best corrected visual acuity (BCVA), intraocular pressure (IOP) measured by Goldman applanation tonometry and OCT measurements were recorded during follow-up. Changes in these parameters with treatment were evaluated and compared with fellow eyes (non-affected eyes) and healthy controls.

Patients with an ocular diagnosis other than cataract or who had previously undergone intraocular surgery were not included in the study. Also, patients with sleep apnea syndrome in their medical records were not included in the study.

Macular and choroidal thickness measurements were made with an OCT device (Retinascan advanced RS-3000, NIDEK, Gamagori, Japan). The choroidal area was evaluated as the area between the retina pigment epithelium and the chorioscleral junction. Total choroidal area (TCA) examined was considered to be 750 μm nasally and temporally from the fovea. Distance from the fovea was measured with the scale of the OCT device. For macular and choroidal thickness measurements, at least 3 measurements were taken and their average was recorded.

Choroidal vascularity index (CVI) was assessed as the ratio of lumen area (LA) to TCA. OCT images were converted to 8-bit format and thresholded using ImageJ (v1.47, NIH) to segment out vascular lumens. Binarization of the choroid followed established methods described in the literature [11]. Each binarized image was converted to RGB, and luminal areas were further isolated using color thresholding. TCA, LA, and stromal area (SA) were subsequently calculated within a central 1500 μm zone, with bright pixels representing the choroidal interstitial space and dark pixels representing the vascular luminal space.

Statistical analyses were performed with IBM SPSS Statistics, Version 23.0 (SPSS Inc., Chicago, USA). Descriptive statistics, frequencies and percentages within the group were reported as (*n*, %). Before analyzing the relationship between continuous variables between groups, they were subjected to normality analyses considering the number of samples in the groups. For this purpose, analytical methods (Kolmogorov-Smirnov and Shapiro-Wilk tests) and visual evaluation methods (histogram graphs) were used. Accordingly, variables with normal distribution were reported as mean ± standard deviation, and variables with non-normal distribution were reported as median (minimum-maximum). Difference analysis between the two groups in terms of numerical variables was performed using the Student’s T test, which compares the means for those with normal distribution, and the Mann-Whitney U test, which compares the median values for those with non-normal distribution. The difference in the distribution of categorical data between groups was evaluated with the Chi-square test. For significance, Pearson Chi-Square or Fisher’s Exact test p values were used, considering the number of patients in the categories.

Comparison of the patient’s eye findings with the fellow eyes was made using the Dependent Sample T test for normally distributed variables, and the Wilcoxon Signed-Rank test for non-normally distributed variables. The change in eye findings over time (baseline, 1st, 3rd and 6th months) was examined using Repeated-Measures ANOVA for normally distributed variables. The Greenhouse-Geisser correction was used in cases where the sphericity assumption was not met. Pairwise comparisons were evaluated using the Dependent Sample T test, with Bonferroni correction made when necessary. For non-parametric repeated measures, the Friedman test was used to find the statistical significance of change over time. If necessary, Bonferroni correction was made and pairwise comparisons were evaluated with the Wilcoxon Signed-Rank test. For statistical significance, the total type-1 error level was determined as 5%.

## Results

Twenty-nine patients diagnosed with RVO and 29 healthy subjects were included in the study. Demographic and clinical characteristics of the groups are shown in Table 1. There was no significant difference between the groups in terms of age and gender (*p* = 0.726, *p* = 0.293 respectively). Body mass index (BMI) was found to be significantly higher in the patient group than in the control group (*p* = < 0.001). Two patients were observed to be pseudophakic. All patients in the control group were phakic.

**Table 1:** Demographic and characteristic features of the groups

	Group 1 (RVO) (*n*:29) mean ± sd	Group 2 (Fellow eyes) (*n*:29) mean ± sd	Group 3 (Control) (*n*:29) mean ± sd	*p* value
Age, years	61.17 ± 8.79	61.17 ± 8.79	61.90 ± 6.75	0.726^¥^
Gender (Male) *n* (%)	17 (58)	17 (58)	13 (44)	0.293*
BMI, kg/m^2^	31.60 ± 5.99	31.60 ± 5.99	25.95 ± 3.17	**< 0.001**
Systemic diseases None HT DM + HT MTHFR gene mutation Parkinson’ disease Asthma HT + CAD Faktör V Leiden Mt.	1 13 8 1 1 1 1 3			
Classification of RVO Superotemporal RVO Inferotemporal RVO Central RVO	19 7 3			
Ischemic RVO Non-ischemic RVO	7 22			

* RVO * retinal vein occlusion, *BMI* body mass index, *HT* hypertension, *DM* diabetes mellitus, *CAD* coronary arter disease

^¥^ İndependent Sample’s T test

*Chi-square test

A comparison of pre-treatment measurements of the affected eyes with the fellow eyes and the control group is shown in Table 2. BCVA was significantly lower in the affected eyes compared to the other two groups (*p* = < 0.001, *p* = < 0.001). Central macular thickness, subfoveal choroidal thickness, SA, and TCA values were found to be significantly higher in the patient group than in the other two groups (*p* = < 0.05 for each). CVI was significantly lower in the patient group compared to the other two groups (*p* = < 0.001, *p* = < 0.001).

**Table 2:** Comparison of clinical features between groups before treatment

	Group 1 (Pre-injection) (*n*:29)	Group 2 (Fellow eyes) (*n*:29)	Group 3 (Control) (*n*:29)	p1	p2	p3
BCVA (Snellen)	0.25 ± 0.19	0.94 ± 0.09	1.00 ± 0.00	**< 0.001**	**< 0.001**	**0.002**
IOP (mmHg)	16.69 ± 3.72	15.52 ± 2.44	16.52 ± 2.93	0.753	0.845	0.923
CMT (µm)	561.14 ± 261.50	232.93 ± 13.93	230.52 ± 14.62	**< 0.001**	**< 0.001**	0.522
SCT (µm)	351.45 ± 44.64	285.52 ± 31.06	278.79 ± 28.29	**< 0.001**	**< 0.001**	0.400
LA (mm^2^)	0.40 ± 0.04	0.35 ± 0.05	0.40 ± 0.06	**< 0.001**	0.623	**0.004**
SA (mm^2^)	0.25 ± 0.03	0.18 ± 0.02	0.18 ± 0.02	**< 0.001**	**< 0.001**	0.494
TCA (mm^2^)	0.63 ± 0.07	0.53 ± 0.08	0.59 ± 0.08	**< 0.001**	**0.003**	**0.023**
CVI	60.66 ± 1.39	66.53 ± 2.28	68.80 ± 1.37	**< 0.001**	**< 0.001**	**< 0.001**

*BCVA* best corrected visual acuity, *IOP* intraocular pressure, *CMT* central macular tickness, *SCT* subfoveal choroid tickness, *LA* lumen area, *SA* stromal area, *TCA* total choroid area, *CVI* choroid vascularity index

p1: Dependent Samples T test, Group 1 vs. Group 2

p2: Independent Samples T Test, Group 1 vs. Group 3

p3: Independent Samples T Test, Group 2 vs. Group 3

The changes in the findings of the patient group at baseline and at the 1st, 3rd, and 6th months after the injection are shown in Table 3. BCVA increased significantly in the 6th month, more in the first month of injection (*p* < 0.05 for each). The ticknesses of the macula and subfoveal choroid, SA and TCA decreased significantly after injection (*p* < 0.05 for each). CVI values increased significantly, especially in the 1st month after injection (*p* < 0.05 for each).

**Table 3:** Findings before and after Intravitreal Injection in the Retinal Vein Occlusion Group

	Pre-injection	Post-injection 1st month	Post-injection 3rd month	Post-injection 6th month	*p* value*
BCVA (Snellen acuity)	0.25 ± 0.19	0.46 ± 0.26	0.55 ± 0.28	0.65 ± 0.29	**0 vs. 1: 0.007** **0 vs. 3: <0.001** **0 vs. 6: <0.001** **1 vs. 3**: 0.451 **1 vs. 6: 0.001** **3 vs. 6**: 0.32
IOP (mmHg)	16.69 ± 3.72	16.03 ± 4.01	16.07 ± 2.90	15.97 ± 1.82	0.662
CMT (µm)	561.14 ± 204.50	348.38 ± 158.52	268.21 ± 71.47	243.10 ± 53.13	**0 vs. 1: 0.003** **0 vs. 3: <0.001** **0 vs. 6: <0.001** **1 vs. 3**: 0.222 **1 vs. 6: 0.007** **3 vs. 6**: 1
SCT (µm)	351.45 ± 44.64	341.03 ± 36.42	292.97 ± 36.03	268.72 ± 36.36	**0 vs. 1: <0.001** **0 vs. 3: <0.001** **0 vs. 6: <0.001** **1 vs. 3: 0.018** **1 vs. 6: <0.001** **3 vs. 6: <0.001**
LA (mm^2^)	0.40 ± 0.04	0.40 ± 0.05	0.40 ± 0.05	0.40 ± 0.05	0.793
SA (mm^2^)	0.25 ± 0.03	0.22 ± 0.02	0.21 ± 0.03	0.19 ± 0.02	**0 vs. 1: <0.001** **0 vs. 3: <0.001** **0 vs. 6: <0.001** **1 vs. 3: 0.01** **1 vs. 6: <0.001** **3 vs. 6: 0.004**
TCA (mm^2^)	0.65 ± 0.07	0.63 ± 0.07	0.61 ± 0.08	0.59 ± 0.07	0 vs. 1: 0.147 **0 vs. 3: 0.002** **0 vs. 6:0.002** **1 vs. 3**: 0.419 **1 vs. 6**: 0.069 **3 vs. 6**: 0.21
CVI	60.66 ± 1.39	64.05 ± 1.97	65.47 ± 1.98	67.24 ± 1.97	**0 vs. 1: <0.001** **0 vs. 3: <0.001** **0 vs. 6: <0.001** **1 vs. 3: 0.003** **1 vs. 6: <0.001** **3 vs. 6: <0.001**

*BCVA* best corrected visual acuity, *IOP* intraocular pressure, *CMT* central macular tickness, *SCT* subfoveal choroid ticknesss, *LA* lumen area, *SA* stromal area, *TCA* total choroid area, *CVI* choroid vascularity index

* “0”= Pre-injection, “1”= Post-injection 1st month, “3”= Post-injection 3rd month, “6”=Post-injection 6th month

Comparison of the findings obtained in the 6th month after intravitreal injection in the patient group with the other two groups is summarized in Table 4. Although significant increase in BCVA was observed in the patient group, it was significantly lower than the other two groups (*p* = < 0.001, *p* = < 0.001). In the patient group, ticknesses of the macula decreased to similar levels in the fellow eye and the control group, no difference was observed between the groups (*p* > 0.05 for each). While no change was observed in LA, CVI values were observed to increase and were at fellow eye levels (*p* = 0.055). There was no significant difference between the groups in terms of IOP values (*p* > 0.05 for each).

**Table 4:** Comparison of clinical characteristics between groups at the 6th Month after Injection

	Group 1 (Post-injection) (*n*:29)	Group 2 (Fellow eyes) (*n*:29)	Group 3 (Control) (*n*:29)	p1	p2	p3
BCVA (Snellen acuity)	0.65 ± 0.29	0.94 ± 0.09	1.00 ± 0.00	**< 0.001**	**< 0.001**	**0.002**
IOP (mmHg)	15.97 ± 1.82	15.52 ± 2.44	16.52 ± 2.93	0.299	0.394	0.923
CMT (µm)	243.12 ± 53.21	232.93 ± 13.93	230.52 ± 14.62	0.312	0.224	0.522
SCT (µm)	268.72 ± 36.36	285.52 ± 31.06	278.79 ± 28.29	**0.014**	0.250	0.400
LA (mm^2^)	0.40 ± 0.05	0.35 ± 0.05	0.40 ± 0.06	**0.004**	0.698	**0.004**
SA (mm^2^)	0.197 ± 0.02	0.180 ± 0.02	0.185 ± 0.02	**0.019**	0.116	0.494
TCA (mm^2^)	0.59 ± 0.07	0.53 ± 0.08	0.59 ± 0.08	**0.006**	0.800	**0.023**
CVI	67.24 ± 1.97	66.53 ± 2.28	68.80 ± 1.37	0.055	**0.001**	**< 0.001**

*BCVA* best corrected visual acuity, *IOP* intraocular pressure, *CMT* central macular tickness, *SCT* subfoveal choroid ticknesss, *LA* lumen area, *SA* stromal area, *TCA* total choroid area, *CVI* choroid vascularity index

p1: Dependent Samples T test, Group 1 vs. Group 2

p2: Independent Samples T Test, Group 1 vs. Group 3

p3: Independent Samples T Test, Group 2 vs. Group 3

The changes in macular and choroidal thickness of occlusive and non-occlusive areas in eyes with BRVO following treatment are shown in Table 5. No significant change was observed in the macular thickness of non-occlusive areas with treatment, while other areas showed a statistically significant decrease.

**Table 5:** Macula and Choroid thicknesses of Occlusive and non-occlusive areas in BRVO patients

	Pre-injection	Post-injection 1st month	Post-injection 3rd month	Post-injection 6th month	*p* value*
Macular tickness, Occlusive area (*n*:26) (µm)	642.12 ± 138.13	489.54 ± 117.02	408.12 ± 104.45	349.19 ± 76.69	**0 vs. 1: 0.027** **0 vs. 3: <0.001** **0 vs. 6: <0.001** **1 vs. 3**: 0.576 **1 vs. 6: <0.001** **3 vs. 6: 0.037**
Macular tickness, Non-occlusive area (*n*:26) (µm)	287.50 ± 18.55	288.88 ± 30.14	284.04 ± 24.55	281.12 ± 24.25	0.272
Choroid tickness, Occlusive area (*n*:26) (µm)	385.38 ± 45.97	337.42 ± 35.52	319.62 ± 35.53	292.88 ± 34.64	**0 vs. 1: < 0.001** **0 vs. 3: < 0.001** **0 vs. 6: < 0.001** **1 vs. 3: 0.005** **1 vs. 6: < 0.001** **3 vs. 6: 0.001**
Choroid tickness, Non-occlusive area (*n*:26) (µm)	305.50 ± 41.81	270.92 ± 27.54	261.92 ± 37.92	246.12 ± 34.21	**0 vs. 1: < 0.001** **0 vs. 3: < 0.001** **0 vs. 6: < 0.001** **1 vs. 3**: 0.416 **1 vs. 6: < 0.001** **3 vs. 6**: 0.082

*BRVO* branch retinal vein occlusion

* “0”= Pre-injection, “1”= Post-injection 1st month, “3”= Post-injection 3rd month, “6”=Post-injection 6th month

## Discussion

The prevalence of RVO increases with age and the 15-year cumulative incidence has been shown to be 2.3% [4]. A complex interplay of systemic and ocular factors contributes to the pathogenesis of RVO. Main accompanying diseases are HT, DM, HL, smoking, atherosclerotic vascular diseases, coagulation disorders, hyperviscosity, vasculitis, and platelet function disorders [5]. The average age in our study group was 61 years, as we know higher age is an important risk factor for RVO. Among identified risk factors for RVO, HT stands out as the most prominent, with its prevalence further underscored by its high frequency within this patient cohort. In the study, the ischemic RVO rate was 24%, and this rate was found to be compatible with the literature [12–14].

Many parameters are used for choroidal evaluation. Although choroidal thickness is the most commonly used method, it is affected by many ocular and systemic factors such as age, gender, diurnal variation, and ethnicity. According to Agrawal, the use of CVI to measure the vascular status of the choroid becomes very important because it is not affected by many of the factors mentioned above [15]. Given the potential limitations associated with choroidal ticknesses, including low reproducibility and reliability, CVI presents as a potentially more stable and objective marker for assessing choroidal vascularity. This approach offers the potential to overcome the shortcomings of relying solely on choroidal tickness measurements. In our study, parameters such as macular and choroidal thickness, CVI, LA, SA, and TCA were examined in RVO cases. Additionally, changes in these parameters were evaluated with preinjection and postinjection follow-up measurements after intravitreal injection were examined.

Although many studies evaluated choroidal thickness in RVO cases, there is no consensus. It has been shown that choroidal thickness is increased in RVO patients compared to contralateral eyes, and there is a decrease in central choroidal thickness after intravitreal dexamethasone implant treatment [16, 17]. Similarly, Tsuiki et al. showed that central choroidal thickness increased in 36 newly developed CRVO cases compared to contralateral eyes [18]. Unlike these results, Du et al. reported that there was no difference in choroidal thickness between eyes with RVO and fellow eyes in their population-based study [19]. We can say that the reason for these differences in the current studies is that Du et al. evaluated chronic RVO cases, unlike the other three studies. In this study where we evaluated acute RVO cases, we found that choroidal thickness was greater in eyes with RVO than in fellow eyes.

Mitamura et al. compared affected and unaffected eyes in 40 patients with newly diagnosed RVO (10 had CRVO and 30 had BRVO) and reported that affected eyes had higher subfoveal choroid tickness, TCA, and SA values [20]. CVI value was lower than the fellow eye, while the LA values were similar. Additionally, they reported that SA and TCA were significantly reduced in affected eyes 1, 3, and 6 months after intravitreal avastin administration in RVO cases. Aribas et al. also reported lower CVI values in sixty-four eyes with unilateral central or branch RVO compared to the healthy eyes and the control group [21]. Hwang et al. showed lower CVI values in 35 patients with monocular BRVO and macular edema than in healthy eyes [22]. In our study, CVI values were found to be lower and subfoveal choroid tickness, TCA, and SA values were higher in affected eyes compared to the fellow eyes and the control group. Differently, while the LA value was significantly higher compared to the fellow eyes, there was no significant difference compared to the control group. After intravitreal avastin injection, a significant decrease was observed in subfoveal choroid tickness, SA and TCA values, in the 1st month, 3rd, and 6th months. CVI increased significantly in the follow-up, more in the 1st month after injection. No change was observed in LA.

While RVO primarily impacts the retina, alterations in the choroid’s structure are likely secondary consequences of the retinal pathology. In our study, we found that the increase in choroidal thickness was mainly due to SA expansion rather than LA, and this expansion of the SA indicates choroidal stromal edema. The increase in SA, unlike other retinal diseases, is particularly noteworthy in RVO cases with macular edema. In central serous chorioretinopathy, there was an increase in only LA throughout the entire choroid, and in diabetic retinopathy, there was an increase in both LA and SA [23]. Therefore, an increase in SA appears to be a specific marker for RVO.

**Fig. 2:**
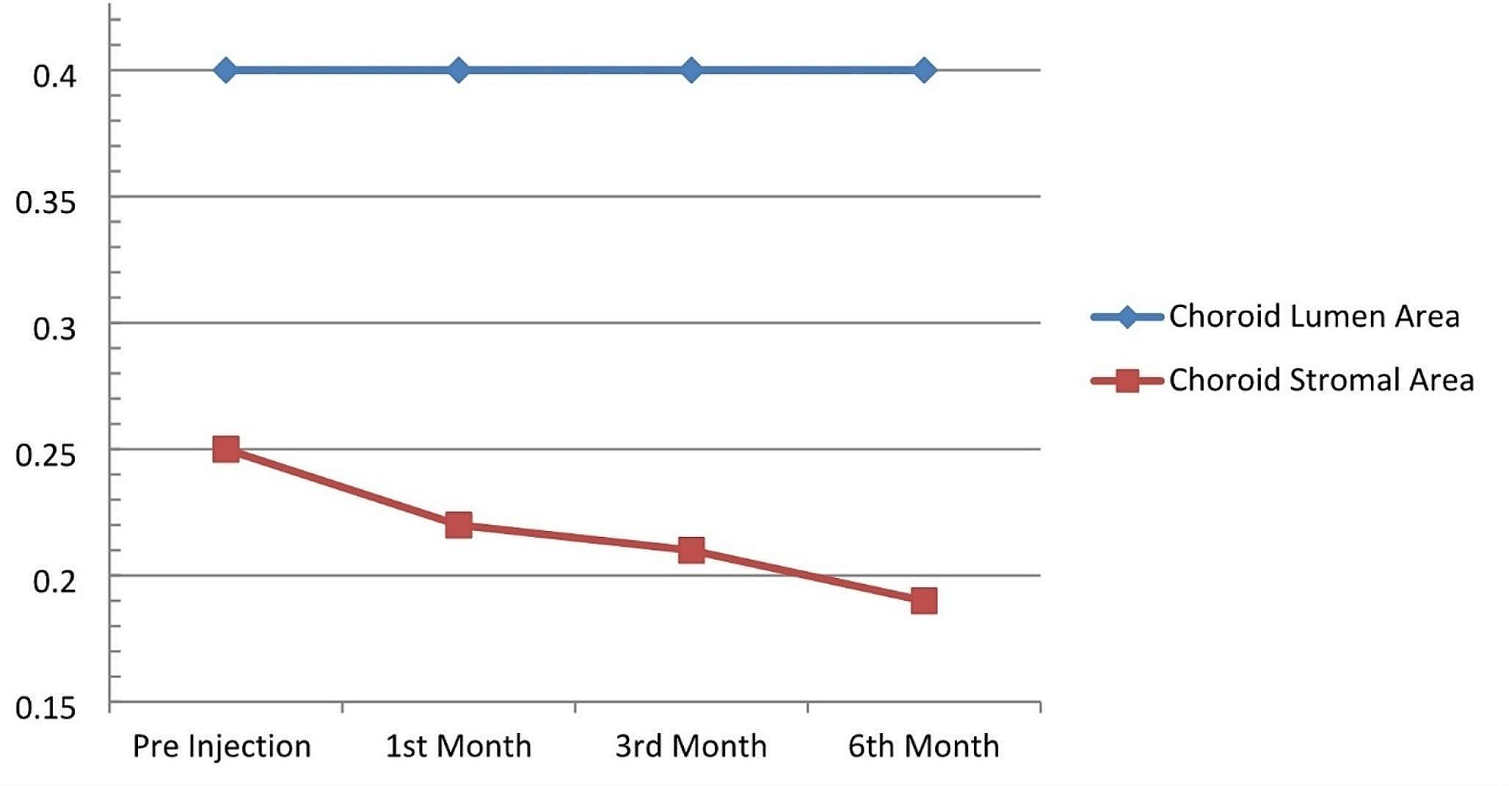
Graph showing the decrease in choroid stromal area without change in lumen area with intra vitreal anti-VEGF treatment in eyes with RVO

Retinal ischemia triggers the production of VEGF, a potent angiogenic factor. This VEGF can migrate to the choroid, stimulating choroidal vascular growth and potentially contributing to thickness increase [8, 24]. As in many studies in the literature, our study showed that choroidal stromal edema normalized with anti-VEGF treatment, supporting the role of VEGF in choroidal thickness. Research using monkeys revealed a notable decrease in the number of fenestrations on choriocapillaris endothelial cells after an intravitreal injection [25]. It can be concluded that such vascular leaks are reduced by anti-VEGF treatment, thereby reducing choroidal stromal edema. Although increased choroidal thickness has been reported in eyes with macular edema due to acute-onset RVO, no increase in choroidal thickness was detected in eyes with long-term RVO and without macular edema [19]. It is assumed that intraocular VEGF concentration does not increase significantly in chronic cases without macular edema [26]. This supports the role of excess VEGF in choroidal edema pathology in acute cases. Although the study by Rayess showed that the initial choroidal thickness was higher than in the healthy eye, when the patients were divided into groups that responded to treatment and those that did not, it was observed that the initial choroidal thickness was higher in those who responded to treatment than in those who did not respond to treatment [27]. In patients who did not respond to treatment, the choroidal thickness was found to be close to that of the healthy eye. This may be attributed to the previously mentioned increase in choroidal thickness associated with high VEGF benefiting more from anti-VEGF treatment (see Fig. 2).

Kim et al. compared 57 BRVO eyes with the fellow eyes and normal controls and reported that the choroidal thickness in the region where vein occlusion developed was significantly greater than the choroid in the subfoveal and non-occlusive regions [28]. They also showed that choroidal thickness in eyes with BRVO correlated with macular edema in both the central and occlusive areas. Our analysis of macular and choroidal thicknesses within both occlusive and non-occlusive areas revealed significantly greater values in the occlusive region. While macular and choroidal thicknesses within the occlusive area exhibited some reduction at 6 months post-intravitreal injection, they remained significantly elevated compared to the non-occlusive region. The observed regional variations in choroidal thickness in RVO may be attributed to direct fluid leakage from occluded vessels into surrounding tissues, suggesting a direct link between RVO and choroidal thickening. The localized extravascular fluid leak and/or elevated hydrostatic pressure observed in RVO may stem from damaged vascular endothelial cells, vasodilatation, and increased blood flow through occluded retinal vessels [28]. Increased vascular permeability, hydrostatic pressure, and VEGF levels within the occluded area led to fluid accumulation as cystoid macular edema and subretinal fluid. Notably, in eyes receiving intravitreal injection, macular thickness in the occluded area significantly decreased, while the non-occlusive area remained unchanged. In addition, the choroidal thickness in the occlusive area reached levels similar to the control group at 6 months after intravitreal injection. These findings indicate that the increase in macular and choroidal thickness in the occlusive area is primarily due to VEGF and will benefit from anti-VEGF treatment. In addition, we can predict that VEGF load is higher in cases with greater choroidal thickness and that they benefit more from anti-VEGF treatment. The significant decrease in the macular and choroidal thickness in the occlusive area in the first month after intravitreal injection also supports this situation.

The small sample size and limited follow-up period can be considered as limitations of this study. Furthermore, investigating both CRVO and BRVO patients separately in larger cohorts may provide more precise answers to specific questions regarding choroidal changes in each subtype. However, the prospective nature of our study and its demonstration of structural changes in the macula and choroid with treatment of the affected eye and fellow eye in RVO patients are its strengths.

## Conclusion

The increased choroidal thickness in eyes with macular edema associated with RVO was primarily caused by SA rather than choroidal LA and decreased after anti-VEGF treatment. In eyes with RVO, LA remains unchanged, SA increases, secondary CVI decreases, and choroidal thickness increases. Contrary to initial assumptions, the observed choroidal thickening in RVO appears primarily driven by stromal edema, likely triggered by elevated intraocular VEGF levels, rather than direct vascular dilatation.
